# Classifications of Neurodegenerative Disorders Using a Multiplex Blood Biomarkers-Based Machine Learning Model

**DOI:** 10.3390/ijms21186914

**Published:** 2020-09-21

**Authors:** Chin-Hsien Lin, Shu-I Chiu, Ta-Fu Chen, Jyh-Shing Roger Jang, Ming-Jang Chiu

**Affiliations:** 1 Department of Neurology, National Taiwan University Hospital, College of Medicine, National Taiwan University, Taipei 100225, Taiwan; chlin@ntu.edu.tw (C.-H.L.); tfchen@ntu.edu.tw (T.-F.C.); 2 Department of Computer Science and Information Engineering, National Taiwan University, Taipei 10617, Taiwan; shui.chiu@mirlab.org (S.-I.C.); jang@csie.ntu.edu.tw (J.-S.R.J.); 3 Department of Computer Science, National Chengchi University, Taipei 11605, Taiwan; 4 Graduate Institute of Biomedical Electronics and Bioinformatics, National Taiwan University, Taipei 10617, Taiwan; 5 Graduate Institute of Brain and Mind Sciences, National Taiwan University, Taipei 100233, Taiwan; 6 Graduate Institue of Psychology, National Taiwan University, Taipei 10617, Taiwan

**Keywords:** Alzheimer’s disease, Parkinson’s disease, frontotemporal dementia, neurodegenerative disorders, biomarkers, deep learning model, linear discriminant analysis, classification, multivariate imputation by chained equations

## Abstract

Easily accessible biomarkers for Alzheimer’s disease (AD), Parkinson’s disease (PD), frontotemporal dementia (FTD), and related neurodegenerative disorders are urgently needed in an aging society to assist early-stage diagnoses. In this study, we aimed to develop machine learning algorithms using the multiplex blood-based biomarkers to identify patients with different neurodegenerative diseases. Plasma samples (*n* = 377) were obtained from healthy controls, patients with AD spectrum (including mild cognitive impairment (MCI)), PD spectrum with variable cognitive severity (including PD with dementia (PDD)), and FTD. We measured plasma levels of amyloid-beta 42 (Aβ42), Aβ40, total Tau, p-Tau181, and α-synuclein using an immunomagnetic reduction-based immunoassay. We observed increased levels of all biomarkers except Aβ40 in the AD group when compared to the MCI and controls. The plasma α-synuclein levels increased in PDD when compared to PD with normal cognition. We applied machine learning-based frameworks, including a linear discriminant analysis (LDA), for feature extraction and several classifiers, using features from these blood-based biomarkers to classify these neurodegenerative disorders. We found that the random forest (RF) was the best classifier to separate different dementia syndromes. Using RF, the established LDA model had an average accuracy of 76% when classifying AD, PD spectrum, and FTD. Moreover, we found 83% and 63% accuracies when differentiating the individual disease severity of subgroups in the AD and PD spectrum, respectively. The developed LDA model with the RF classifier can assist clinicians in distinguishing variable neurodegenerative disorders.

## 1. Introduction

According to the World Health Organization (WHO), the global population is aging and the number of people over 60 years old is expected to rise from 900 million in 2015 to more than 2 billion in 2050. As populations have aged, the incidence and prevalence of common neurodegenerative disorders, such as Alzheimer’s disease (AD) and Parkinson’s disease (PD), have risen [1]. AD is the most important cause of dementia in the elderly population; its pathological hallmarks are intraneuronal tau accumulations as neurofibrillary tangles and extracellular amyloid plaques depositions. Older adults can progress to having mild cognitive impairment (MCI) without it affecting their daily activities, but further cognitive decline deteriorates daily function as AD dementia [2]. The second most common dementia is frontotemporal dementia (FTD), which is also characterized by intraneuronal phosphorylated tau depositions. FTD is often hard to differentiate from AD in a clinical diagnosis, especially in the early stage of the disease. The α-synuclein that contains Lewy body accumulation in dopaminergic neurons of substantia nigra is the pathological characteristic of PD [3] and loss of dopamine results in progressive motor dysfunction. Notably, PD patients deteriorate not only in their motor aspects but also in cognitive function, which is defined as PD with dementia (PDD) [4]. Currently, there is no cure for neurodegenerative diseases like FTD, AD, and PD, and their diagnoses, which require a combination of clinical assessments, neuropsychological testing, neuroimaging, and exclusion of other neurological disorders, most commonly occur when clinical symptoms are developed during significant underline disease progress [5,6]. Moreover, objective monitoring of disease progression is hampered by the lack of suitable markers.

Recently suggested AD diagnoses are brain positron emission tomography (PET) scans, which can detect amyloid and tau depositions, and cerebrospinal fluid (CSF) analysis, which can detect candidates for pathological proteins [7]. However, these relatively invasive and costly procedures may limit their clinical usefulness in a large-scale aged population. Given the likely entry of several classes of mechanism-targeted therapies for mitigating neurodegeneration in AD or PD into early human clinical trials [8,9], identifying easily accessible blood-based biomarkers to reflect disease severity is urgently needed. These neuropathology-related proteins, including amyloid-beta 42 (Aβ42), Aβ40, total Tau, phosphorylated Tau, and α-synuclein, are present in human body fluids, including CSF and blood plasma [10,11], which are good candidates for surrogate biomarkers for disease severity in patients with AD and PD. However, overlapping neuropathology findings have been found in patients with AD and PD. For example, pathological studies have also identified amyloid plaques and tau-containing neurofibrillary tangles as hallmark pathologies of AD in patients’ post-mortem brains with PDD [12] and FTD [13]. These overlapping pathology findings suggest that a single molecule biomarker is not sufficient as a disease-specific biomarker or a marker for monitoring disease progression as mixed pathology may exist in patients with advanced stage diseases.

Our group has previously detected increased plasma levels of Aβ42, total and p-tau T181 in patients with AD, and increased plasma α-synuclein in patients with PD compared to controls using an immuno-magnetic reduction method (IMR) [10,11,14]. However, classifying different neurodegenerative disorders is difficult, especially in early disease stages. A single-molecule detection in plasma may miss a group of patients with mixed pathology, and strategies simultaneously analyzing the neuropathology-related biomarker candidates in plasma, combined with deep learning algorithms, are needed. Therefore, we aimed to develop a machine learning-based model using multiplex blood-based biomarker information collected from participants of normal aging, AD or PD spectrum, and FTD to identify patients in the early stage of these diseases. We conducted such a model in the hopes that patients may benefit from a future clinical trial of disease-modifying therapeutics to mitigate neurodegeneration and monitor disease progression.

## 2. Results

### 2.1. Clinical Characteristics

Table 1 summarizes the demographic data of participants and their five biomarkers’ plasma levels, including Aβ42, Aβ40, total Tau, p-Tau181, and α-synuclein for all 377 individuals. Neurologically normal healthy controls (*n* = 97), patients on the AD spectrum (including mild cognitive impairments (MCI, *n* = 41) and AD (*n* = 35)), patients on the PD spectrum (including PD patients with normal cognition (*n* = 57)), PD patients with MCI (*n* = 29) and PDD (*n* = 87), and FTD (*n* = 31), were enrolled in the current study. The age and disease duration were significantly higher in patients with AD and PDD than in other patient groups and in controls (*p* < 0.01 by ANOVA). Women were more prevalent on the AD spectrum, and men were more prevalent on the PD spectrum. The mini mental state examination (MMSE) scores were significantly lower in patients on the AD, PDD, and FTD groups than other groups and controls (*p* < 0.01 by ANOVA). Patients with PDD had significantly greater motor severity (Hohen–Yahr stages) compared with PD with normal cognition, or the PD-MCI group (*p* < 0.01 by ANOVA).

### 2.2. Plasma Biomarker Levels in Different Disease Groups

**Figure 1:**
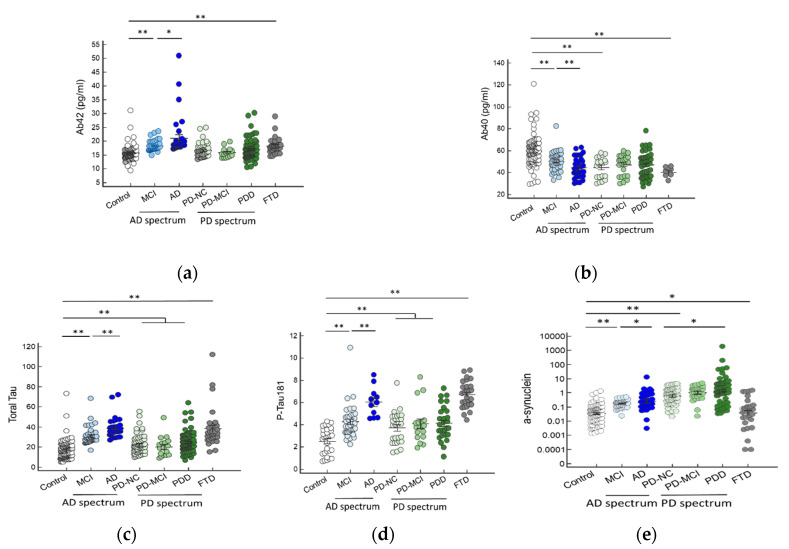
Individual plasma biomarker levels of normal controls and in different disease groups. The plasma Aβ42 (**a**) and Aβ40 (**b**) levels significantly increased in patients with AD and FTD, especially when compared to the normal control and other disease groups (*p <* 0.01). The plasma total tau (**c**) and *p*-tau181 (**d**) level significantly increased in patients with FTD and then followed by AD, MCI, and PDD group (*p <* 0.01). The plasma α-synuclein (**e**) levels were highest in the PDD group than other disease groups and controls. The mean ± one standard deviation (SD) was illustrated as horizontal lines in each disease group. * *p <* 0.05; ** *p <* 0.01.

We compared individual biomarker levels in different disease groups. We found that plasma levels of Aβ42 were increased in patients on the AD spectrum, whereas FTD patients compared to the controls (Figure 1a). Among the AD spectrum groups, Aβ42 levels were significantly higher in patients with AD than in patients with MCI (21.15 ± 7.17 vs. 18.30 ± 1.85, *p* < 0.01). The levels of Aβ40 were lower in patients on the AD spectrum groups, PD with normal cognition, and FTD compared to the controls (Figure 1b). The Aβ40 levels were lower in the AD group than patients with MCI (44.73 ± 9.47 vs. 49.01 ± 21.10, *p* < 0.01). The plasma levels of total Tau and *p*-Tau181 were significantly higher in patients on the AD spectrum groups, PD spectrum groups, and FTD patients, with the highest level in the FTD group (Figure 1c,d). The plasma α-synuclein levels were increased in all disease groups, except FTD, when compared to controls (Figure 1e). The α-synuclein levels were highest in patients with PDD. The changes in plasma biomarkers of amyloid and Tau were indistinguishable between the FTD and the AD groups.

### 2.3. The LDA Model for Classifying Controls, AD Spectrum, PD Spectrum, and FTD

**Figure 2:**
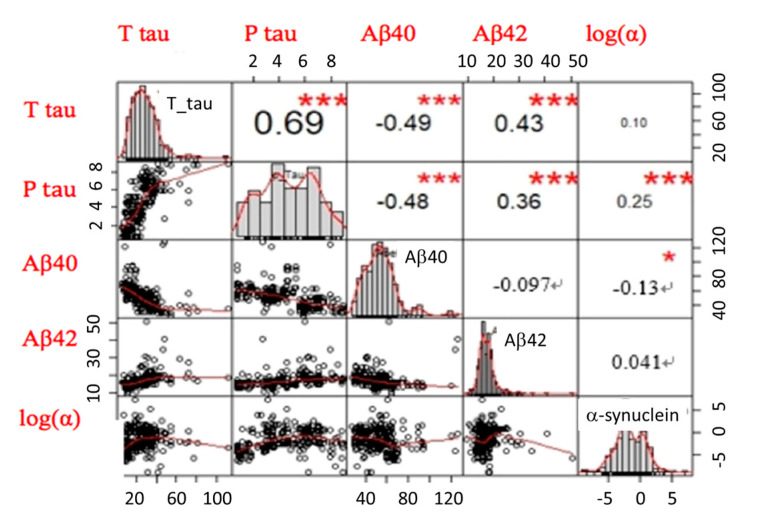
The correlation matrix between each biomarker from four groups, including healthy controls, as well as patients with Alzheimer’s disease (AD) spectrum, Parkinson’s Disease (PD) spectrum, and Frontotemporal Dementia (FTD). The upper triangular part of the matrix is the correlation coefficients between any two biomarkers. The lower triangular part of the matrix is the scattered-plot graphs of any two biomarkers. The main diagonal part of the matrix is the distribution graphs of each biomarker. log(α) is the log of α-synuclein. * *p <* 0.05; *** *p <* 0.001.

We applied a linear discriminant analysis (LDA) to reduce the data input from 5 biomarker features of 377 participants into 2-dimensional or 3-dimensional information to classify individual dementia groups (i.e., AD spectrum, PD spectrum, and FTD). The purpose of LDA is to find the best linear combinations of the 5-biomarker features to separate each group with the highest accuracy. Among these three dementia groups, differentiating the pattern of 5 biomarkers between AD and FTD was crucial because single individual marker levels were comparable between these two groups. We then performed a visualization of scatter plots of each participant’s data in a 2-dimensional or 3-dimensional model. Such reductions can sometimes lead to a better classification accuracy since it can avoid the curse of dimensionality. We successfully reduced the 5-dimensional data from 5 biomarkers’ information to a 3-dimensional model using the correlation matrix between each marker, as shown in Figure 2. * *p <* 0.05; ** *p <* 0.01.

**Figure 3:**
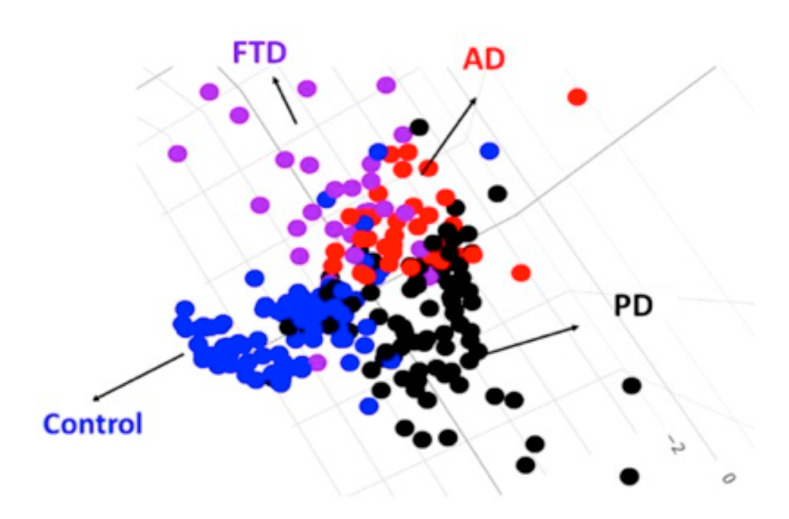
The 3D scatter plot demonstrates that LDA separated sample points of different dementia groups. AD: AD spectrm, PD: PD spectrum.

The established 3-dimensional model was based on the linear discriminant function, as shown below, to illustrate the samples’ 3D scatter plot and to accurately classify the three dementia groups and controls (Figure 3).

*f = 0.01408Tau + 0.26819pTau − 0.0040Aβ40 + 0.06062Aβ42 + 0.37553log(α − synuclein)* (1)

The *f* is the best discriminant function of linear combinations of the original 5-biomarker features to separate each group.

### 2.4. The LDA Model for Classifying Controls and AD Spectrum

**Figure 4:**
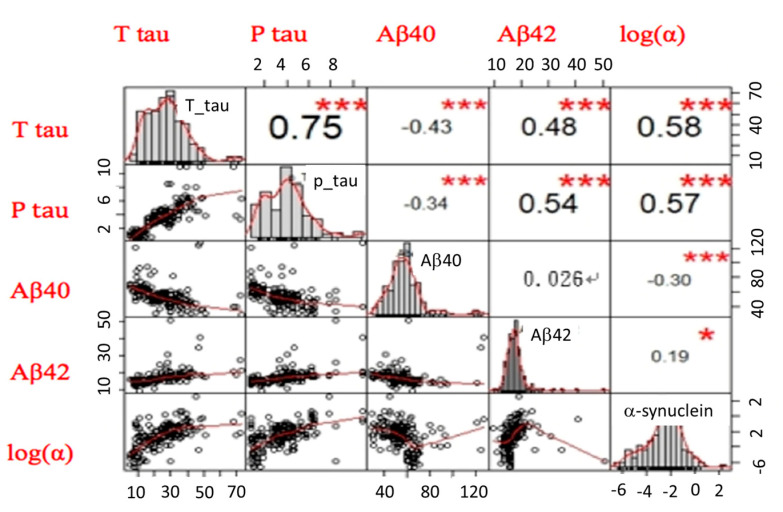
The correlation matrix between each biomarker from three groups, including normal controls, patients with mild cognitive impairment (MCI), and AD. The upper triangular part of the matrix is the correlation coefficients between any two biomarkers. The lower triangular part of the matrix is the scattered-plot graphs of any two biomarkers. The main diagonal part of the matrix is the distribution graphs of each biomarker. log(α) is the log of α-synuclein. * *p <* 0.05; *** *p <* 0.001.

Among patients on the AD spectrum, we applied the same approach to reduce the dimensionality from 5 dimensions to 2 dimensions to sperate patients with AD from MCI using the correlation matrix between each marker, as shown in Figure 4.

**Figure 5:**
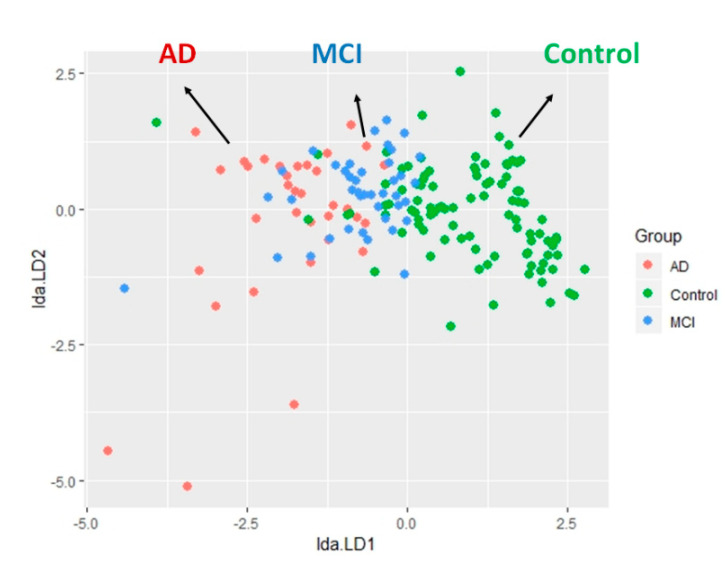
The 2D scatter plot demonstrates that LDA separated sample points of the AD spectrum.

The established 2-dimensional model was based on the linear discriminant function, as shown below, to illustrate the 3D scatter plot of the samples and to accurately classify the patients with AD from MCI, and the controls (Figure 5).

*f = −0.04303Tau − 0.22985pTau + 0.0075712Aβ40 − 0.087502Aβ42 − 0.23726log(alpha_synuclein)* (2)

### 2.5. The LDA Model for Classifying Controls and PD Spectrum

**Figure 6:**
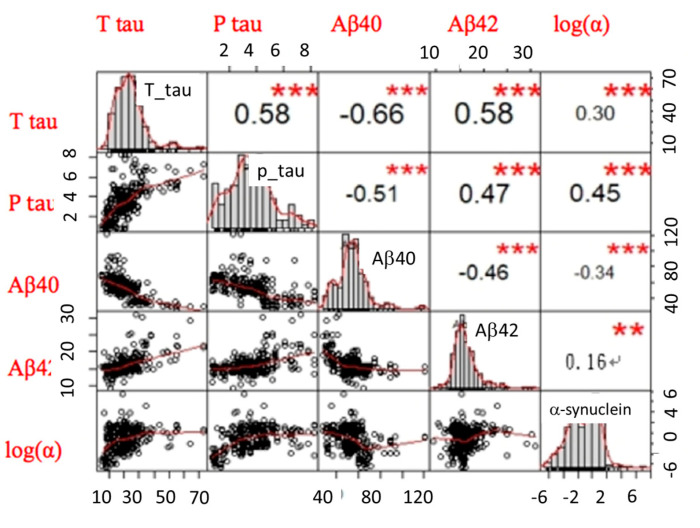
The correlation matrix between each biomarker from four groups, including normal controls, PD-NC, PD-MCI, and PDD. The lower triangular part of the matrix is the scattered-plot graphs of any two biomarkers. The main diagonal part of the matrix is the distribution graphs of each biomarker. log(α) is the log of α-synuclein. ** *p <* 0.01; *** *p <* 0.001.

Among patients on the PD spectrum, we applied the same approach to reduce the dimensionality from 5 dimensions to 3 dimensions to sperate individual groups of PD patients, including PD with normal cognition (PD-NC), PD with MCI (PD-MCI), and PDD, using the correlation matrix between each marker, as shown in Figure 6.

**Figure 7:**
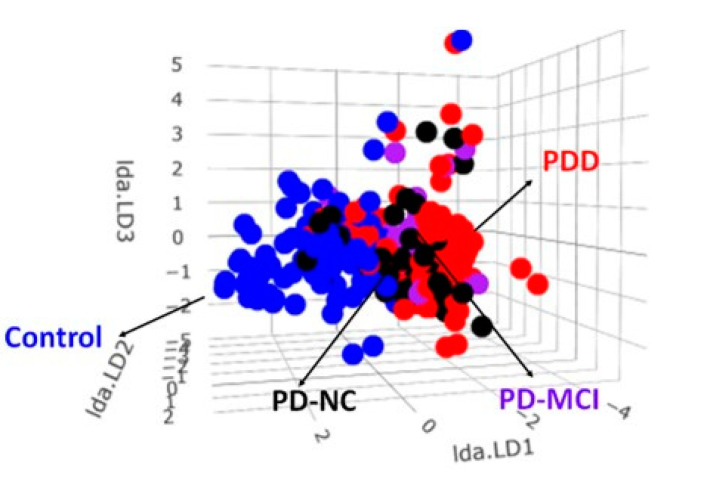
The 3D scatter plot demonstrates that LDA separated sample points of different subgroups of the PD spectrum.

The established 3-dimensional model was based on the linear discriminant function, as shown below, to illustrate the 3D scatter plot of the samples and classify the patients with normal controls, PD-NC, PD-MCI, and PDD (Figure 7).

*f = −0.00489Tau−0.156621pTau + 0.006844 Aβ40 + 0.027807 Aβ42 − 0.57530 log(α-synuclein)* (3)

### 2.6. Measure the Performance of the Established Models by LDA

To measure the performance of the established three models based on LDA’s selected features, we used 7 benchmark deep-learning classifiers to predict the accuracy. These classifiers include the Naïve Bayes (NB) [15], k-Nearest Neighbor (kNN) [16], support vector machine (SVM) [17], C4.5 decision tree (C4.5) [18], classification and regression trees (CART) [19], random forest (RF) [20], and logistic regression (LogReg). A good feature selection method should have high learning accuracy but less computational overhead (i.e., time complexity and space complexity). We then used the leave-one-out cross-validation (LOOCV) method to objectively evaluate our model construction procedure [21].

**Figure 8:**
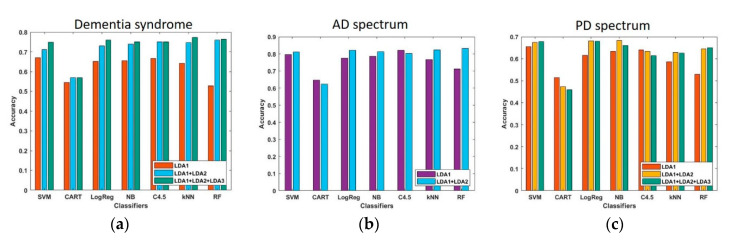
The estimated accuracy for models classifying different dementia syndrome (**a**), AD spectrum (**b**), and PD spectrum (**c**). LDA1: the first function of the linear combinations based on the original five biomarker features using linear discriminant analysis; LDA2: the second function of the linear combinations based on the original five biomarker features using linear discriminant analysis; LDA3: the third function of the linear combinations based on the original five biomarker features using linear discriminant analysis.

We found that the RF was the best algorithm to classify each of the different dementia syndromes (i.e., AD, PDD, and FTD), with an accuracy rate of 3 transformed features in the respective axis of the 3D-model over 0.76 (Figure 8a). For the AD spectrum (MCI and AD), the accuracy was measured by different classifiers and is shown in Figure 8b. RF provided the highest accuracy rate of 0.83 with two transformed features in the individual axis of the established 2D-model. For the PD spectrum (PD-NC, PD-MCI, and PDD), the accuracy is shown in Figure 8c, with the highest accuracy rate reaching up to 0.68.

## 3. Discussion

The results of this study demonstrated that integrated plasma biomarkers combined with deep-learning models could be applied to classify normal aging controls from patients with different spectrums of neurodegenerative diseases. Furthermore, the established models simultaneously incorporate information from five disease-pathology related biomarkers, which could provide a better classification between different disease severity on the AD and PD spectrums. Several machine-learning-based approaches were used in this study to classify different disease groups. We use MICE for imputing missing data; LDA was applied for dimensionality reduction and feature extraction to show the samples’ biomarker information as a 2D or 3D scatter plot for better visualization of separation. Moreover, several deep-learning algorithms were employed to examine the established models’ accuracy, and the RF classification system was found to have the best performance for accuracy. These developed models with multiplex biomarker information could help clinicians distinguish diseases in their early-stages and reflect disease severity on the AD and PD spectrums.

We previously used the IMR method to establish platforms to detect plasma levels of disease-related proteins, including Aβ42, Aβ40, total Tau, p-Tau181, α-synuclein, and p-α-synuclein Ser129 [10,11,14,22]. The two hallmark pathologies of AD are the extracellular Aβ plaque deposits and the flame-shaped neurofibrillary tangles of the microtubule-binding protein tau. Our findings revealed that in this current mixed neurodegenerative population, Aβ42 plasma levels increased in patients with AD compared to MCI and controls. Besides, Aβ42 plasma levels were higher in MCI than those in controls compatible with a previous study, suggesting that Aβ42 plasma levels can differentiate healthy control from subjects with MCI [23]. From a pathological point of view, Aβ deposition plateaus when patients progress into the clinical MCI phase of AD at the time of cognitive symptoms [24]. On the contrary, Aβ40 levels decreased in AD patients compared to MCI and controls. Our results were in line with recent findings that plasma Aβ peptide ratio (Aβ42/Aβ40) predicts brain amyloid-β-positive or negative status via amyloid-β-PET imaging [25]. Previous studies have utilized the IMR method that demonstrates an increase in plasma Aβ42 in AD patients compared to controls, which correlated negatively with the CSF levels of Aβ42 [26,27]. The amyloid plaques in the post-mortem brain pathology of AD patients mainly consists of Aβ42, although Aβ40 is more abundant than Aβ42 in the brain and plasma [28]. Oligomers form readily from the Aβ42 peptide but much less from the more abundant Aβ40 [29]. The C-terminus of Aβ42 is critical for oligomer formation. There is a close correlation between the ratio of Aβ42/Aβ40 and the age of disease onset in familial AD [30]. Our findings in the plasma may reflect the increased aggregative Aβ42 in the AD disease process. Another hallmark of AD, tau, was also increased in the plasma and CSF of AD patients [31].

Further studies have shown a strong correlation between plasma p-tau181 with Tau PET, and a high concordance with CSF p-Tau levels [32,33]. These findings indicate that plasma levels of total Tau and p-Tau combined with the Aβ42/ Aβ40 ratio could be a surrogate marker for AD. On the other hand, another dementia syndrome, FTD, which was characterized by neuronal tau accumulations, showed increased levels of total Tau and p-Tau181 rather than the Aβ42/Aβ40 ratio. Furthermore, patients with PDD revealed increased levels of α-synuclein rather than other marker proteins, compared to those with PD-MCI and PD with normal cognition. These results suggested that higher levels of plasma α-synuclein are associated with poorer cognitive performance in PD patients. This association supports Braak’s hypothesis that cortical Lewy body/neurotic pathology is more extensive in PDD than in PD without dementia [3]. However, differentiating neurodegenerative disorders is challenging [34], especially in the early disease stages. Pathologically dementia with Lewy bodies (DLB) and PDD cannot be easily distinguished; both diseases may show concomitant AD pathology, especially in older individuals; however, this is more commonly observed in DLB. It has even found that cortical and striatal Aβ depositions are virtually always present in DLB [35]. The concurrence of multiple biomarkers such as Aβ and tau abnormalities and alpha-synuclein suggests different proteinopathies may add specificity of underlying pathology to mixed dementia.

In addition to targeting these disease pathology-related proteins, several groups have adopted an unbiased approach, including proteomics, metabolomics, and gene expression profiling [36,37,38]. However, most of these previous studies are limited to relatively small sample size or have had difficulty replicating their findings [39]. Therefore, we developed a machine learning-based model that used plasma biomarker data collected from 377 participants experiencing normal aging, on the AD or PD spectrums, and FTD to predict and differentiate different neurodegenerative disorders. Machine learning algorithms are broadly applied to support healthcare systems, i.e., early diagnosing, precision medicine, and genetic screening [40]. Recently, an aptamer-based technology (SOMAmer assay, SomaLogic) combined with an RF deep-learning classification system was used to measure 1047 proteins in three tissue types from PD patients and controls (e.g., serum, CSF, post-mortem brain tissues). The results showed that testing the serum samples offered promising results with an AUC (area under the receiver operating characteristic curve) of 0.77 [41]. Moreover, a recent study that applied a typical approach of training machine learning algorithms using the public gene database from 160 AD and 127 healthy controls produced models with an average sensitivity of 48.7% (95% CI = 34.7–64.6) [42]. Our study applied LDA to reduce dimensionality and extract features from the multiplex blood biomarkers and then distinguished individual disease subgroups using the RF classifier, which provided an average accuracy of 76% for the AD and PD spectrums, as well as FTD. Moreover, accuracies of 83% and 63% were found when differentiating individual disease severity subgroups on the AD and PD spectrums, respectively. Future studies should combine other markers, including neuroimages and genetic risk factors, and are needed to polish the model to further classify and predict individual neurodegenerative disorders in the early-stage or prodromal stage of the disease process.

This study used the multiplex biomarker information from various patients with the most common neurodegenerative disorders and age/gender-matched healthy controls, which provided more comprehensive data about plasma levels of disease-related pathology proteins than just assaying a single marker. Overlapping neuropathology was found in patients with AD and PD, and even in PDD. These overlapping pathology findings suggest the need for an integrated multiple biomarker panel, which incorporates a novel strategy combing suitable data processing and deep-learning algorithm to identify surrogate biomarker information for assessing the risk and monitoring the progression of neurodegenerative disorders.

However, our study has several limitations. First, most of the patients diagnosed with AD, PD, or FTD already receive medication treatments. As therapeutic drugs may affect plasma protein profiling, such as memantine (a common drug used to treat AD symptoms) [43], the established classification models may have inadvertently learned protein expression perturbations due to treatment rather than disease biology. Therefore, it would fail in the clinical setting to diagnose AD or PD patients who are naïve to medication. Second, the mean age of our controls is younger than those in the AD spectrum and PDD. Age may influence the expressions of targeted proteins in the plasma. Plasma levels of the total Tau and Aβ42 levels have modest but significant correlations with chronological age [44] while there is no significant correlation between age and plasma α-synuclein levels in neurologically healthy controls [11,22]. This age effect was not considered in our machine-learning algorithm, and future classification models incorporating age effects are warranted. Another limitation is the lack of inclusion of the TDP-43 biomarker in this report. FTD consists of a spectrum of clinical syndromes associated with several underlying neurodegenerative diseases characterized by frontotemporal lobar degeneration (FTLD) [45] and neuropathologically, most (90–95%) FTLDs are caused by intracellular aggregates of p-tau or TAR DNA-binding protein 43 (TDP-43) [46]. Finally, the clinical diagnosis was not confirmed neuropathologically and is therefore susceptible to misclassification. However, the final diagnosis was based on thorough clinical and ancillary investigations (including nuclear imaging and neuropsychological assessment) after extensive clinical follow-up and following international consensus criteria in specialized memory or movement disorder clinics. Large-scale cohort studies with a long-term follow-up combined with drugs and co-morbidity information are needed to validate our results.

In conclusion, our study used information from the 5-disease pathology-related plasma biomarkers from 377 patients with various neurodegenerative disorders and age/gender-matched controls. We explored several classification models using deep learning algorithms and found that the RF classifier can best help clinicians distinguish patients with different neurodegenerative diseases and monitor their progression. Future validation in a large-scaled heterogeneous aging population is needed to confirm our findings. A future application of this integrated approach combing with multi-domain markers, including structural brain MRI or molecular PET images and biomarkers in other biofluids, will assist identification of disease even at the earliest asymptomatic pre-clinical stage. In this context, patients would benefit most in the pre-clinical stage from this biomarker-guided intervention, which could provide the best chance to mitigate neurodegeneration.

## 4. Materials and Methods

### 4.1. Study Participants

All participants were recruited from the memory or movement disorder clinics in the National Taiwan University Hospital (NTUH), a tertiary referral center in Taiwan. We analysed 377 plasma samples from patients with MCI (*n* = 41), AD (*n* = 35), PD with normal cognition (PD-NC, *n* = 57), PD with mild cognitive impairment (PD-MCI, *n* = 29), PDD (*n* = 87), FTD (*n* = 31), and age/gender-matched healthy controls (*n* = 97). MCI and AD were diagnosed according to the National Institute on Aging–Alzheimer’s Association (NIA-AA) workgroup for clinical diagnosis of MCI and AD [47]. PD was diagnosed according to the United Kingdom PD Society Brain Bank clinical diagnostic criteria [48]. PD-MCI was diagnosed according to the Movement Disorder Society (MDS) task force diagnostic criteria using the level I global cognitive function test [49]. MDS task force criteria also were used to diagnose PDD, with an MMSE score of 25 or less as the cut-off for identifying significant cognitive impairment in PD patients, as well as impairment of instrumental activities of daily living (e.g., inability to manage finances and cope in social situations) [4]. This study was approved by the National Taiwan University Hospital’s institutional ethics board committee (201406125DSC, 20160470 MINC, NTUH 201903094RINA). All participants or their proxy provided written informed consent to participate in the study.

### 4.2. Biomarker Assessments

A total of 10 mL of venous blood was drawn from each participant and centrifuged (2500× *g* for 15 min) within 3 h of collection. The plasma levels of Aβ42, Aβ40, total Tau, phosphorylated Tau (p-Tau181), and **α-**synuclein were measured via IMR methods, as previously described [11,14,27].

### 4.3. Statistical Analyses for Clinical and Biomarker Characteristics

Numerical variables are expressed as means ± standard deviations or medians with 95% confidence intervals (CIs). For variables following a Gaussian distribution, data were compared using two-tailed *t*-tests, and multiple comparisons were performed using analysis of variance (ANOVA). For variables not following a normal distribution, data were compared using the Mann–Whitney test, which is the non-parametric equivalent of the independent samples t-test, and the Kruskal–Wallis test was used for comparing three or more groups. We performed all analyses with Stata (StataCorp LP, College Station, TX, USA) software. A *p* value of <0.05 was considered significant.

### 4.4. Data Processing and Dimensionality Reduction

For some missing values of biomarker data in the dataset due to the plasma samples’ suboptimal quality, we used multivariate imputation by chained equations (MICE) to perform data imputation [50]. Moreover, we performed the following two data adjustment operations to make the dataset more compliant for machine learning. First, the values of α-synuclein were transformed into the logarithm function for their ultra-low levels in the plasma. Second, we put each biomarker feature into a linear min-max normalization. Therefore, each feature had a minimum value of 0 and a maximum value of 1 to make each feature have a similar distribution range.

In statistics and machine learning, dimensionality reduction is the process of reducing the number of features such that the characteristics of the reduced dataset can be retained as much as possible. Approaches of dimensionality reduction can be divided into feature selection and feature extraction. In the current study, we employed LDA to perform dimensionality reduction and feature extraction. We visualized scatter plots in 2D or 3D. Such reduction can sometimes lead to a better classification accuracy since it avoids the effects of the curse of dimensionality.

**Figure 9:**
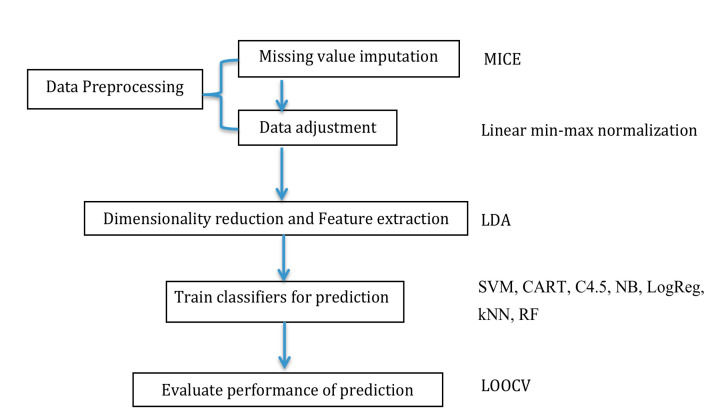
Data processing flow chart in this study; MICE: multivariate imputation by the chained equation; LOOCV: leave-one-out cross-validation; NB: naïve Bayes; *k*NN: k-nearest neighbor; SVM: support vector machine; C4.5: C4.5 decision tree; CART: classification and regression trees; RF: random forest; LogReg: logistic regression.

We used 7 deep-learning classifiers (i.e., SVM, CART, C4.5, NB, LogReg, *k*NN, and RF) to compare the accuracy of multiclass classification in individual models. We also used the leave-one-out cross-validation (LOOCV) method to objectively estimate the performance of our model construction procedure [21]. LOOCV is essentially an estimate of a model’s generalization performance trained on n−1 samples of data, which is generally a slightly pessimistic estimate of the performance of a model trained on all n samples. The workflow of the abovementioned data preprocessing is illustrated in Figure 9.

## Figures and Tables

**Table 1:** Clinical characteristics and plasma biomarker levels of study participants in individual groups.

	Controls (*n* = 97)	AD Spectrum		PD Spectrum			FTD (*n* = 31)	*p* Value
		(*n* = 76)		(*n* = 173)				
		MCI	AD	PD-NC	PD-MCI	PDD		
		(*n* = 41)	(*n* = 35)	(*n* = 57)	(*n* = 29)	(*n* = 87)		
Age (years)	64.0 ± 7.8	72.9 ± 7.9	75.2 ± 11.6	62.4 ± 11.2	66.5 ± 11.8	72.8 ± 8.9	60.7 ± 7.1	*p <* 0.01 **
Gender (M, n, %)	31 (31.9%)	16 (39.0)	14 (40.04%)	31 (54.4)	20 (68.9)	53 (60.9)	6 (19.4)	*p <* 0.01 **
Disease duration (y)	N.A.	2.7 ± 0.9	6.2 ± 2.9	2.6 ± 1.1	5.6 ± 1.6	9.6 ± 3.2	5.4 ± 2.1	*p <* 0.01 **
MMSE	28.9 ± 0.9	26.8 ± 1.2	18.2 ± 5.8	28.3 ± 0.9	26.8 ± 1.1	20.8 ± 4.3	18.6 ± 4.8	*p <* 0.01 **
Hoehn-Yahr stage	N.A.	N.A.	N.A.	1.7 ± 0.9	2.0 ± 0.8	2.5 ± 1.1	N.A.	*p <* 0.01 **
Aβ42 (pg/mL)	15.66 ± 2.58	18.30 ± 1.85	21.15 ± 7.17	16.56 ± 2.44	15.80 ± 1.75	16.96 ± 3.54	18.26 ± 2.84	*p <* 0.01 **
Aβ40 (pg/mL)	59.45 ± 13.94	50.28 ± 8.69	49.01 ± 21.10	44.73 ± 9.47	47.10 ± 9.21	46.96 ± 11.93	40.34 ± 10.69	*p <* 0.01 **
Total tau (pg/mL)	16.95 ± 9.61	30.93 ± 8.67	38.70 ± 9.74	22.32 ± 9.91	20.27 ± 10.01	24.71 ± 10.41	38.16 ± 10.73	*p <* 0.01 **
Phospho-tau (pg/mL)	2.52 ± 1.17	4.28 ± 1.51	6.04 ± 1.33	3.72 ± 1.43	4.09 ± 1.77	4.15 ± 1.51	6.69 ± 1.19	*p <* 0.01 **
a-synuclein (pg/mL)	0.09 ± 0.05	0.20 ± 0.11	0.80 ± 0.32	1.15 ± 0.09	1.62 ± 0.28	25.17 ± 7.83	0.05 ± 0.02	*p <* 0.01 **

** *p <* 0.01.

## Abbreviations

MCI	Mild cognitive impairment
AD	Alzheimer’s disease
PD	Parkinson’s disease
PD-NC	Parkinson’s disease with normal cognition
PD-MCI	Parkinson’s disease with mild cognitive impairment
FTD	Frontotemporal dementia
LDA	Linear discriminant analysis
MICE	Multivariate imputation by chained equation
LOOCV	Leave-one-out cross-validation
NB	Naïve Bayes
kNN	k-nearest neighbor
SVM	Support vector machine
C4.5	C4.5 decision tree
CART	Classification and regression trees
RF	Random forest
LogReg	Logistic regression.
